# Bioactivity-Guided Purification of Novel Herbal Antioxidant and Anti-NO Compounds from *Euonymus laxiflorus* Champ.

**DOI:** 10.3390/molecules24010120

**Published:** 2018-12-30

**Authors:** Van Bon Nguyen, San-Lang Wang, Anh Dzung Nguyen, Zhi-Hu Lin, Chien Thang Doan, Thi Ngoc Tran, Hung Tse Huang, Yao-Haur Kuo

**Affiliations:** 1 Institute of Research and Development, Duy Tan University, Da Nang 550000, Vietnam; 2 Department of Chemistry, Tamkang University, New Taipei City 25137, Taiwan; doanthng@gmail.com (C.T.D.); tranngoctnu@gmail.com (T.N.T.); 3 Life Science Development Center, Tamkang University, New Taipei City 25137, Taiwan; 4 Institute of Biotechnology and Environment, Tay Nguyen University, Buon Ma Thuot 630000, Vietnam; 5 Division of Chinese Materia Medica Development, National Research Institute of Chinese Medicine, Taipei 11221, Taiwan; tiger77749@gmail.com; 6 Department of Science and Technology, Tay Nguyen University, Buon Ma Thuot 630000, Vietnam; 7 Division of Chinese Materia Medica Development, National Research Institute of Chinese Medicine, Taipei 11221, Taiwan; kk49310953@nricm.edu.tw; 8 Department of Biochemical Science and Technology, National Taiwan University, Taipei 106, Taiwan; 9 Graduate Institute of Integrated Medicine, College of Chinese Medicine, China Medical University, Taichung 40402, Taiwan; 10 Ph.D. Program for Clinical Drug Development of Chinese Herbal Medicine, College of Pharmacy, Taipei Medical University, Taipei 11031, Taiwan

**Keywords:** antioxidants, anti-NO, *Euonymus laxiflorus*, Walterolactone A (1a) and B (1b) β-d-glucopyranoside, bioactive compounds

## Abstract

*Euonymus laxiflorus* Champ., a medicinal herb collected in Vietnam, has been reported to show several potent bioactivities, including anti-NO, enzyme inhibition, hypoglycemic and antidiabetic effects. Recently, the antioxidant activity of *Euonymus laxiflorus* Champ. trunk bark (ELCTB) has also been reported. However, the active antioxidant and anti-NO constituents existing in ELCTB have not been reported in the literature. The objective of this study was to purify the active antioxidants from ELCTB and investigate the anti-NO effect of the major constituents. Twenty-two phenolics isolated from ELCTB, including 12 compounds newly isolated in this study (**1**–**12**) and 10 constituents obtained from our previous work, were evaluated for their antioxidant activity. Of these, 12 compounds (**4**–**6**, **9**, **13**–**15**, **18**–**22**) showed a potent antioxidant capacity (FRS50 = 7.8–58.11 µg/mL), in comparison to α-tocopherol (FRS50 = 23 µg/mL). In the anti-NO activity tests, Walterolactone A (1a) and B (1b) β-d-glucopyranoside (**13**) demonstrated the most effective and comparable activity to that of quercetin with max inhibition and IC_50_ values of 100%, 1.3 µg/mL, and 100%, 1.21 µg/mL, respectively. The crude extract and its major compounds showed no cytotoxicity on normal cells. Notably, three constituents (**9**, **11**, and **12**) were identified as new compounds, another three constituents, including **1**, **7**, and **8**, were found to be new natural products, constituents **9** and **13** were determined to be new antioxidants, and compound **13** was reported to have novel potent anti-NO activity for the first time. The results of this study contribute to the enrichment of new natural products and compounds, as well as the novel biological activity of constituents isolated from *Euonymus laxiflorus* Champ. The current study also indicates ELCTB as a rich natural source of active phenolics. It is suggested that ELCTB could be developed as a health food with promising antioxidant and anti-NO effects, as well as other beneficial biological activities.

## 1. Introduction

Living organisms are negatively affected by free radicals in areas such as DNA, proteins, and lipid damage, and as such, they may cause various diseases [1]. These unexpected effects can be reduced and prevented by antioxidant compounds which can be synthesized or obtained from natural resources. However, synthetic antioxidants can cause carcinogenesis and liver damage [2]. Thus, the discovery of abundant and natural antioxidants is still needed. Antioxidants may be obtained from several natural sources, including higher plants [3,4,5], microbial fermentation [6,7], and marine products [8,9]. Of these, herbal antioxidants have been given much exploration in recent years [10].

Vietnam is a tropical country and is considered the sixteenth most biodiverse region in the world. It possesses around 10,000 herbal species, of which 4000 have been used as medicinal plants [11]. Therefore, there has been increased interest in the seeking and purification of bioactive constituents from Vietnamese herbs in recent years [4,5,10,12,13,14,15,16,17]. Numerous herbs showing enzymic inhibitory, hypoglycemic, and antidiabetic effects have been investigated [4,5,12,13,14,15,16,17]. The antioxidant and anticancer effects of the herbs collected in this biodiverse region have also been reported in several works [4,5,10]. However, only a few herbs possessing anti-NO properties have been reported, and there are also few studies working on the isolation and identification of active compounds (antioxidant and anti-NO constituents). Thus, the investigation of natural products processing beneficial properties from the herbs of this biodiverse area has been received with great attention.

*Euonymus laxiflorus* Champ.—A medicinal herb, is wildly distributed in Vietnam [12,17] and some other Asian countries, including China, Cambodia, India, and Myanmar [18]. This medicinal herbal species has been recognized as a rich source of bioactive natural products and has been extensively investigated to reclaim its potent antidiabetic properties via enzymic inhibition and animal tests [4,12,13]. These previous studies revealed that *Euonymus laxiflorus* Champ. shows potency for α-amylase inhibition [13], α-glucosidase inhibition [12], as well as a significant effect on the reduction of blood glucose in diabetic rats [4]. Recently, novel potent antidiabetic compounds have been isolated and identified from *Euonymus laxiflorus* trunk bark extract [14,17]. Of these, poly condensed tannin, a major antidiabetic compound, has demonstrated a significant effect on reducing plasma glucose in ICR mice [17]. Some constituents isolated from the leaves of this plant have shown anti-nitric oxide and antioxidant effects [19]. However, little data on the antioxidant properties of this herbal extract exists beyond the previous reports [4], and no compounds isolated from the trunk bark extract of this herb have been reported for the evaluation of antioxidant and anti-NO capacities.

As a part of the objective to indicate scientific proof for the development of *Euonymus laxiflorus* Champ. as potent functional food or drug, this study investigated the bioactivity of this herb to isolate and identify its chemical constituents. In the current study, the potent antioxidant capacity of *Euonymus laxiflorus* Champ. was reclaimed, and the isolation and identification of its active compounds, as well as an evaluation of its antioxidant and anti-NO activities, were also reported. The results of this study contributed to the enrichment of new natural products and novel compounds, as well as the new biological effects of the constituents isolated from *Euonymus laxiflorus*.

## 2. Results and Discussion

### 2.1. Reclamation of Euonymus laxiflorus Champ. Extracts as a Potential Source of Beneficial Bioactive Properties without Toxicity

**Figure 1:**
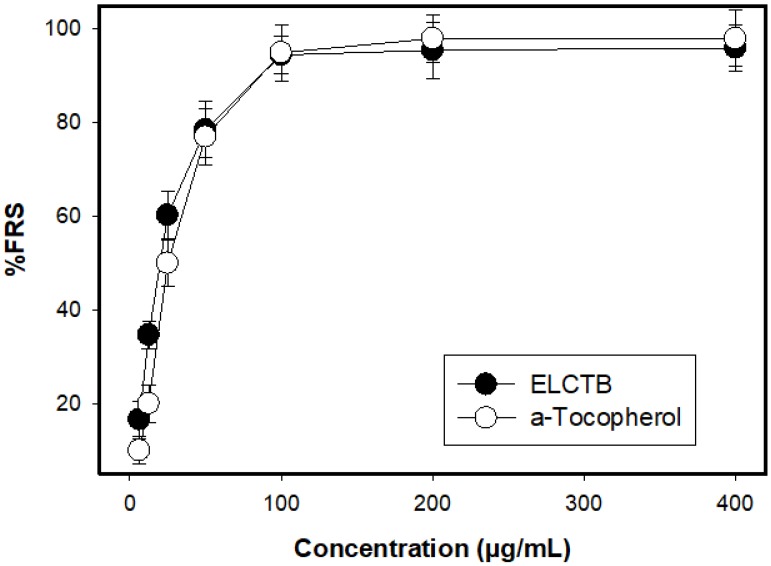
Antioxidant activity (%) of the methanol extract of *Euonymus laxiflorus* Champ. trunk bark (ELCTB) and α-tocopherol. The methanol extract of ELCTB and α-tocopherol were tested at their concentration range of 3.125–400 µg/mL. FRS: Free radical scavenging

The methanol extract of *Euonymus laxiflorus* Champ. trunk bark (ELCTB) was evaluated for its DPPH radical scavenging activity (antioxidant activity), and the results are illustrated in Figure 1 (%) and Table 1 (FRS50). ELCTB showed potent antioxidant activity with great max activity (%) and low FRS50 values of 97% and 20.2 µg/mL, respectively. The antioxidant activity of α-tocopherol, a commercial antioxidant compound, was also tested for comparison and showed approximately equal activity, with max activity (%) and low FRS50 values of 99% and 24.4 µg/mL, respectively. In the comparison, *Euonymus laxiflorus* Champ. also showed comparable or higher antioxidant activity than those of some recently-reported medicinal extracts collected in the central highlands of Vietnam (Table 1).

The trunk bark of *Euonymus laxiflorus* Champ. has been reported to possess promising α-glucosidase inhibitory and α-amylase inhibitory activities [12,13] and significant effects on the reduction of plasma glucose in normal and diabetic mice [4,17]. The anti-NO activity of the extract from the leaves of this herb was investigated by Liu et al. (2014) [19]. In addition, *Euonymus laxiflorus* has been proved to be non-toxic via animal models in previous reports [4]. In this study, ELCTB was found to show potential antioxidant activity, therefore an investigation was conducted to isolate and identify its active compounds, which were subsequently evaluated for their beneficial bioactivities.

### 2.2. Selective Solvent Extraction for Max Antioxidant Productivity from Euonymus laxiflorus Champ. Extracts and Purification of Active Compounds

**Figure 2:**
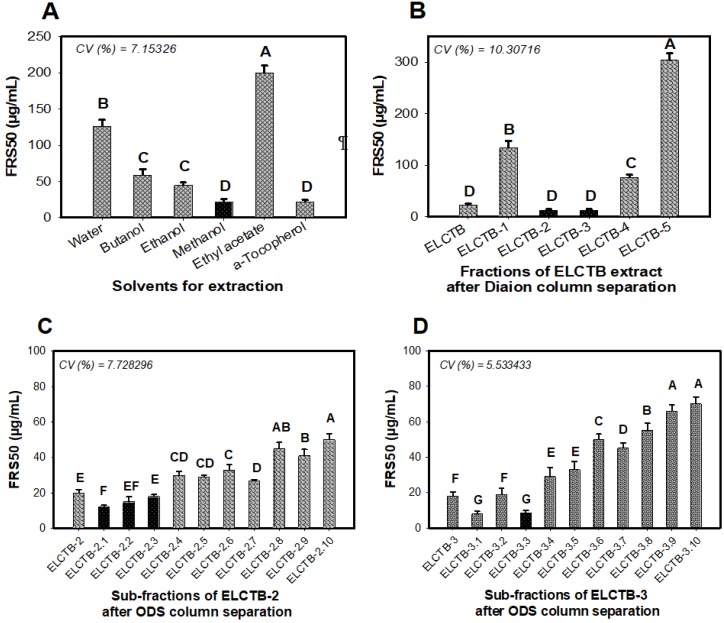
Antioxidant activity of the *Euonymus laxiflorus* Champ. trunk bark (ELCTB) extracted using different solvents: (**A**) ELCTB and its fractions after Diaion column; (**B**) fraction ELCTB-2 and its sub-fractions; (**C**) fraction ELCTB-3 and its sub-fractions; (**D**) after silica columns. The means of FRS50 with different letters in the same figure were significantly different based on Duncan’s multiple range test at alpha = 0.01.

Five solvents, including water, butanol, ethanol, methanol, and ethyl acetate, were used to extract *Euonymus laxiflorus* Champ. trunk bark (ELCTB), and then all extracts were evaluated for antioxidant activity. As shown in Figure 2A, the ELCTB extracted by methanol demonstrated the highest activity due to its smallest FRS50 value of 20 µg/mL. Therefore, the methanol extract was chosen for the next step of purification.

**Figure 3:**
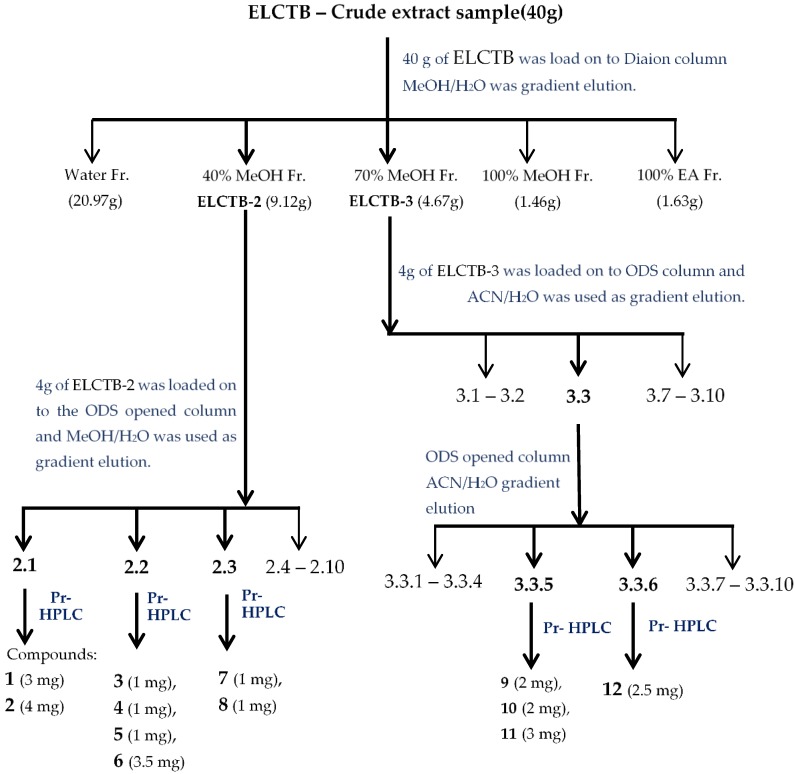
Isolation chart of the active constituents from *Euonymus laxiflorus* Champ. trunk bark (ELCTB) extract. EA: acetyl acetate; MeOH: methanol; Pr-HPLC: preparative high-performance liquid chromatography; ACN: acetonitrile; ODS: octadecylsilane.

ELCTB was primary separated via a Diaion column to obtain the five fractions of ELCTB-1 (20.97 g), ELCTB-2 (9.12 g), ELCTB-3 (4.67 g), ELCTB-4 (1.46 g), and ELCTB-5 (1.63 g) through successive eluting with distilled water, 40% MeOH, 70% MeOH, 100% MeOH, and 100% ethyl acetate, respectively. The antioxidant activity of ELCTB and these separated fractions were tested as shown in Figure 2B. Two fractions (ELCTB-2, and ELCTB-3) showed the smallest FRS50 values of 12.25 and 12.42 µg/mL, respectively, and as such possessed the highest activity. These potent fractions were further purified via ODS columns to obtain a total of 20 sub-fractions. Of these, three sub-fractions, ELCTB-2.1, ELCTB-2.2, and ELCTB-2.3 were separated from ELCTB-2, and one sub-fraction, ELCTB-3.3, was separated from ELCTB-3. These sub-fractions possessed significant activity, and as such were purified to isolate their constituents. The utilization of preparative HPLC resulted in the isolation of 12 (**1**–**12**) compounds from these four active sub-fractions. The isolation process is briefly presented in Figure 3.

### 2.3. Identification of Isolated Constituents from Euonymus laxiflorus Champ.

The 12 constituents newly isolated from ELCTB were identified as Umbelactone (**1**) [20], Walterolactone (**2**) [21], Phenylalanine (**3**) [22], 2-methoxy-4-hydroxyphenol-1-*O*-β-d-glucopyranoside (**4**) [23], 1-β-d-glucopyranosyloxy-3,5-dimethoxy-4-hydroxybenzene (**5**) [24], (−)-3,4-Dihydroxybenzoic acid (**6**) [25], 2-benzoyl myo-inositol (**7**) [26], 1-*O*-Benzoyl-myo-inositol (**8**) [27], walterolactone A/B 6-*O*-gallate-β-d-glucopyranoside (**9**), Roseoside (6S, 9S) (**10**) [28], (3R*,6R*)-tetrahydro-6-ethenyl-2,2,6-trimethyl-*2*H-pyran 3-*O*-α-l-arabinopyranosyl (1→3)-β-d-glucuronopyranosyl (**11**), and 7-Hydroxy-6,7-dihydro-cis/trans- geraniate, 3-*O*-α-l-arabinopyranosyl (1→6)-β-d-glucopyranosyl (**12**).

Based on the literature review, three compounds, Umbelactone (**1**), 2-benzoyl myo-inositol (**7**), and 1-*O*-Benzoyl-myo-inositol (**8**) were found to be new natural products. Notably, walterolactone A/B 6-*O*-gallate-β-d-pyranoglucoside (**9**), (3R*,6R*)-tetrahydro-6-ethenyl-2,2,6-trimethyl-*2*H-pyran 3-*O*-α-l-arabinopyranosyl (1→3)-β-d-glucuronopyranosyl (**11**), and 7-Hydroxy-6,7-dihydro-cis/trans-geraniumsaeure 3-*O*-α-l-arabinopyranosyl (1→6)-β-d-glucopyranosyl (**12**) were identified as new compounds. Their characteristics and NMR data were recorded as shown below. The results of this study contributed to the enrichment of new natural products, as well as the enrichment of the novel constituents of *Euonymus laxiflorus* Champ.

**Figure 4:**
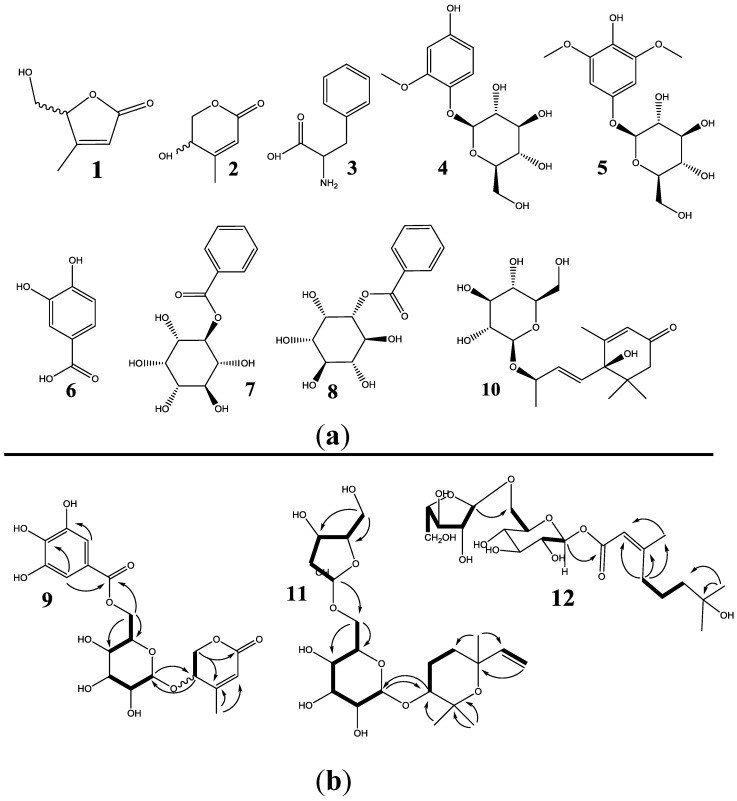
Chemical structures of isolated compounds from the trunk bark of *Euonymus laxiflorus* Champ: (**a**), key correlations of HMBC (heteronuclear multiple bond correlation) and COSY (correlated spectroscopy) of the three new compounds; (**b**) Umbelactone (**1**), Walterolactone (**2**), Phenylalanine (**3**), 2-methoxy-4-hydroxyphenol-1-*O*-β-d-glucopyranoside (**4**), 1-β-d-glucopyranosyloxy-3,5-dimethoxy-4-hydroxybenzene (**5**), 3,4-Dihydroxybenzoic acid (**6**), 2-benzoyl myo-inositol (**7**), 1-*O*-Benzoyl-myo-inositol (**8**), walterolactone A/B 6-*O*-gallate-β-d-glucopyranoside (**9**), Roseoside (6S, 9S) (**10**), (3R*,6R*)-tetrahydro-6-ethenyl-2,2,6-trimethyl-*2*H-pyran 3-*O*-α-l-arabinopyranosyl (1→3)-β-d-glucuronopyranosyl (**11**), and 7-Hydroxy-6,7-dihydro-cis/trans- geraniate, 3-*O*-α-l-arabinopyranosyl (1→6)-β-d-glucopyranosyl (**12**).

*Walterolactone A/B 6-*O*-gallate-β-d-pyranoglucoside* (**9**) was obtained as a white amorphous powder. ^1^H-NMR data (600 MHz, MeOH-*d*_4_, *δ*_H_ ppm): 7.05 (s, 2H), 5.81 (brs), 4.58 (dd, *J* = 12.0, 2.4 Hz), 4.54 (dd, *J* = 12.6, 3.0 Hz), 4.44 (d, *J* = 7.8 Hz), 4.39 (dd, *J* = 12.6, 3.0 Hz), 4.22 (t, *J* = 3.0 Hz), 4.10 (dd, *J* = 12.0, 6.0 Hz), 3.60 (m), 3.4 (m, 2H), 3.2 (m), 2.14 (brs). ^13^C-NMR data (150 MHz, MeOH-*d*_4_, *δ*_C_ ppm): 168.2, 166.0, 158.2, 146.5, 140.0, 121.4, 118.9, 110.1, 103.3, 77.8, 75.6, 74.7, 71.7, 71.6, 70.6, 64.6, 20.5. The chemical structure and the key correlations of HMBC and COSY for this compound are shown in Figure 4.

(3R*,6R*)-tetrahydro-6-ethenyl-2,2,6-trimethyl-2H-pyran 3-*O*-α-l-arabinopyranosyl (1→3)-β-d-glucuronopyranosyl (**11**) was obtained as a white amorphous powder. ^1^H-NMR data (600 MHz, MeOH-*d*_4_, *δ*_H_ ppm): 5.95 (dd, *J* = 18.0, 11.2 Hz), 5.05 (d, *J* = 18.0 Hz), 4.97 (d, *J* = 1.8 Hz), 4.95 (d, *J* = 11.2 Hz), 4.32 (d, *J* = 7.8 Hz), 4.00 (dd, *J* = 11.4, 2.4 Hz), 3.98 (dd, *J* = 3.0, 1.8 Hz), 3.96 (td, *J* = 5.4, 3.0 Hz), 3.82 (dd, *J* = 6.0, 3.0 Hz), 3.74 (dd, *J* = 14.4, 3.0 Hz), 3.64 (dd, *J* = 14.4, 5.4 Hz), 3.60 (dd, *J* = 11.4, 6.0 Hz), 3.43 (m), 3.41 (overlapped), 3.32 (overlapped), 3.23 (dd, *J* = 9.6, 9.0 Hz), 3.12 (dd, *J* = 9.0, 7.8 Hz), 2.15 (ddd, *J* = 13.8, 3.0, 2.4 Hz), 1.97 (dq, *J* = 13.2, 4.2 Hz), 1.75 (qd, *J* = 13.2, 3.6 Hz), 1.60 (td, *J* = 13.8, 2.4 Hz), 1.25 (s, 3 H), 1.18 (s, 3 H), 1.08 (s, 3 H). ^13^C-NMR data (150 MHz, MeOH-*d*_4_, *δ*_C_ ppm): 147.4, 111.5, 109.9, 106.3, 86.0, 85.9, 83.2, 79.0, 78.1, 77.1, 76.6, 75.3, 74.9, 72.0, 68.2, 63.1, 33.6, 32.2, 30.0, 25.8, 22.0. The chemical structure and key correlations of HMBC and COSY for this compound are shown in Figure 4.

*7-Hydroxy-6,7-dihydro-cis/trans-geraniate, 3-*O*-α-l-arabinopyranosyl (1→6)-β-d-glucopyranosyl* (**12**). ^1^H-NMR data (400 MHz, MeOH-*d*_4_, *δ*_H_ ppm): 5.76 (brs), 5.47 (d, *J* = 8.0 Hz), 4.90 (brs), 3.99 (dd, *J* = 11.4, 2.4 Hz), 3.98 (m), 3.96 (m), 3.80 (m), 3.74 (dd, *J* = 11.4, 3.0 Hz), 3.62 (dd, *J* = 11.4, 5.4 Hz), 3.59 (dd, *J* = 11.4, 6.0 Hz), 3.57 (m), 3.42 (t, *J* = 8.8 Hz), 3.34 (m, 2H), 2.20 (t, *J* = 6.8 Hz), 2.18 (s, 3 H), 1.57 (m, 2H), 1.43 (m, 2H), 1.17 (s, 6H). ^13^C-NMR data (100 MHz, MeOH-*d*_4_, *δ*_C_ ppm): 166.4, 164.7, 115.9, 110.0, 95.1, 85.7, 83.2, 78.8, 78.0, 77.5, 73.9, 71.6, 71.2, 67.9, 63.0, 44.0, 42.4, 29.2, 23.2, 19.1. The chemical structure and the key correlations of HMBC and COSY for this compound are shown in Figure 4.

### 2.4. Evaluation of Antioxidant Activity of Identified Compounds from ELCBT

All of the newly identified compounds (**1**–**12**) in this study, as well as the ten compounds (**13**–**22**) obtained from our previous study [14], including walterolactone A (1a) and B (1b) β-d-glucopyranoside (**13**), gallic acid (**14**), (−)-Gallocatechin (**15**), 1-(4-hydroxyphenyl)-2,3-dihydroxypropan-1-one 3-*O*-β-d-glucopyranoside (**16**), Myo-inositol 1-*O*-3,3-dimethylacrylate (**17**), leonuriside (**18**), (+)-Catechin (**19**), methyl galloate (**20**), (−)**-**Catechin (**21**), and (3,5-dimethoxy-4-hydroxyphenol)-1-*O*-β-d-(6′-*O*-galloyl)-glucopyranoside (**22**) were evaluated for their antioxidant capacity at a concentration range of 0.5–250 μg/mL. The activity was estimated and expressed as FRS50 values and the max activity was recorded at 250 μg/mL (Table 2). Of these 22 tested constituents, **6**, **9**, **14** and **19**–**22** demonstrated stronger activity (FRS50 = 7.8–16.77 µg/mL, ranked at the ***e***–***f*** level) than that of α-tocopherol (FRS50 = 23 µg/mL, ranked at the ***d*** level); **13** and **18** showed comparable activity (FRS50 = 27.47–28 µg/mL, ranked at the ***e***–***f*** level) to that of the positive control, since their FRS50 values were ranked at the same level as ***cd***–***d***; while **4**, **5**, and **15** had lower activity (FRS50 = 30.73–58.11 µg/mL, ranked at the ***a***–***c*** level) than α-tocopherol, based on Duncan’s multiple range test at alpha = 0.01. However, these 12 antioxidants all possessed strong max antioxidant activity (92–100%, ranked at the *a* level) that was equal to that of α-tocopherol (99%, ranked at the *a* level); as such they could be identified as potent antioxidant compounds. Compounds **11** and **12** possessed weak activity with max activity of 20–25%, and the other nine constituents showed no activity. The results indicated that ELCBT was a potentially rich source of active antioxidant compounds.

In recent reports, compounds **13**–**15**, **18** and **19** have been reported to show potent α-amylase inhibitory activity [14], and compounds **13**, **15**–**19** were recently determined to be effective α-glucosidase inhibitors by Nguyen et al., 2018 [17]. Of these, Walterolactone A (1a) and B (1b) β-d-glucopyranoside (**13**) was identified as a new compound in a previous study [14]. This study was the first to report its potent antioxidant activity; as such, Walterolactone A (1a) and B (1b) β-d-glucopyranoside (**13**), together with walterolactone A/B 6-*O*-gallate-β-d-glucopyranoside (**9**), which is a new compound investigated in this study, were determined as new effective antioxidants.

### 2.5. Anti-Inflammation and Cytotoxicity of Euonymus laxiflorus Champ. Extract and its Major Compounds

**Figure 5:**
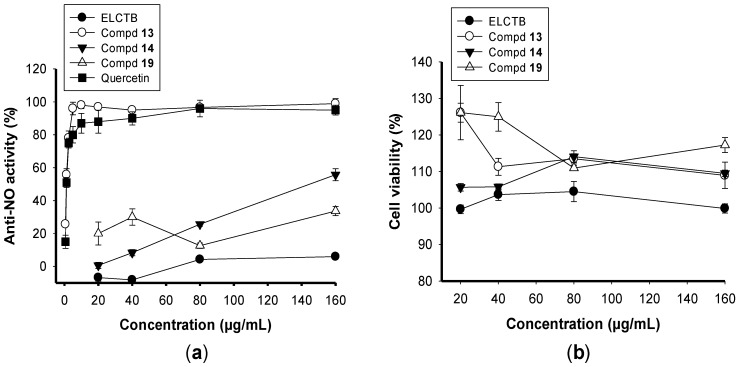
Anti-NO activity (**a**), and cytotoxicity (**b**) of *Euonymus laxiflorus* Champ. trunk bark (ELCTB) extract and its isolated compounds, including Walterolactone A (1a) and B (1b) β-d-glucopyranoside (**13**), gallic acid (**14**), and (+)-Catechin (**19**).

Nitric oxide (NO) has been suggested as a mediator of pro-inflammatory activity related to certain inflammatory disorders, including chronic hepatitis, pulmonary fibrosis, and rheumatoid arthritis. In this study, the crude extract (ELCTB) and its major identified compounds (**1**, **2**, and **15**) were evaluated for anti-NO activity with the use of LPS-stimulated RAW 264.7 cells. As shown in Figure 5a, Walterolactone A (1a) and B (1b) β-d-glucopyranoside (**13**) showed the most effective anti-inflammation properties and gallic acid (**14**) demonstrated acceptable activity with max inhibition and IC_50_ values of 100%, 1.3 µg/mL and 56%, 145 µg/mL, respectively, while (+)-Catechin (**19**) and the crude extract possessed weak activity with a max inhibition of 33.7% and 6%, respectively. Quercetin, a commercial anti-NO compound, was tested for comparison and showed max inhibition and IC_50_ values of 100%, 1.21 µg/mL, respectively. The results indicated that Walterolactone A (1a) and B (1b) β-d-glucopyranoside (**13**) demonstrated promising activity which was equal to that of the positive control. Notably, Walterolactone A (1a) and B (1b) β-d-glucopyranoside (**13**) was investigated for the efficacy of its anti-NO activity for the first time in the current study and was found to be a new effective anti-NO compound. In the previous study, a total of 11 constituents were isolated and identified from the stems and leaves of *Euonymus laxiflorus.* Of these purified compounds, laxifolone A (a triterpene) displayed significant anti-NO effects [29].

The cytotoxicity of *Euonymus laxiflorus* Champ. extract and its major compounds were also examined using the same cell model based on LPS-stimulated RAW 264.7 cells. The cytotoxicity of the crude extract and its major identified compounds were tested using a large concentration range of 20–160 µg/mL. The results (Figure 5b) showed that the major isolated compounds of ELCTB all slightly enhanced the growth of normal cells and that the ELCTB extract, as well as its major compounds (**13**, **14**, and **19**), had no toxicity to normal cells. In the previous study [3], The ELCTB extract was reported to show no toxic effects on a mouse model at a high dose of 300 mg/kg bw (body weight). The results of this study, as well as those of recently reported researches [3], confirmed that ELCTB was a natural and safe material with promising biomedical activities, including enzymic inhibition, hypoglycemic effects, and antidiabetic, antioxidant, and anti-NO activities.

## 3. Materials and Methods

### 3.1. Materials

The ELCBT extract was obtained according to a previous study reported by Nguyen et al., 2017 [11]. 2-diphenyl-1-picrylhydrazyl (DPPH) and ODS gel were purchased from Sigma Chemical Co. (St. Louis City, MO, USA) and Merck Sigma Chemical Co. (St. Louis City, MO, USA), respectively. Reagents, other common chemicals, and solvents were available at the highest grade.

### 3.2. Biological Activity Assays

Free radical scavenging activity was determined through use of the DPPH radical scavenging activity assay described by Nguyen et al., 2018 [9] with minor modifications. One hundred and twenty microliters (120 µL) of the samples (diluted with methanol at a concentration range of 0.5–250 μg/mL) were mixed with 30 µL of 0.75 mM DPPH in methanol in a 96-well plate and then kept in the dark for 30 min. The optical density of the mixtures at 517 nm (OD_517nm_) was measured [30]. The following formula (1) was used to calculate the antioxidant activity:

(1) $$\mathrm{Activity}\;(\%)\;=\;\left(\frac{A-B}{A}\right)\;\times \;100\%$$

where, *A* refers to the OD_517nm_ of the blank sample (no antioxidants/samples in the mixture solution) and *B* stands for the OD_517nm_ of the sample solution at 30 min of incubation. The concentration of an antioxidant compound that can reduce 50% of the purple color of the DPPH solution under the assay conditions was defined as the FRS50 value [9]. α-Tocopherol dissolved in MeOH was used as the positive control.

An anti-inflammation assay was also performed according to the methods described in detail by Nguyen et al., 2018 [9]. The tests were all repeated in triplicate and the differences between the mean values of the antioxidant and anti-NO activity (*p* < 0.01) were analyzed using Statistical Analysis Software (SAS-9.4, provided by SAS Institute Taiwan Ltd., Minsheng East Road, Section 2, Taipei, Taiwan 149-8).

### 3.3. Purification Procedures and Identification of Active Compounds

A total of 40 g of ELCTB extract were loaded onto a Diaion (Mitsubishi Chemical Co., Tokyo, Japan) column and primarily separated into five major fractions: ELCTB-1 (20.97 g), ELCTB-2 (9.12 g), ELCTB-3 (4.67 g), ELCTB-4 (1.46 g), and ELCTB-5 (1.63 g) using successive eluting with distilled water, 40% MeOH, 70% MeOH, 100% MeOH, and 100% ethyl acetate, respectively. ELCTB-2 (4 g) and ELCTB-3 (4 g) were sub-fractionated via the ODS column to obtain 10 sub-fractions (ELCTB.2.1–ELCTB.2.10) as well as 10 sub-fractions (ELCTB.3.1–ELCTB.3.10) eluted with gradient mobile phases of 0–100% MeOH in H_2_O, *v*/*v* and 10–100% ACN in H_2_O, *v*/*v*, respectively. Of these, ELCTB.2.1, ELCTB.2.2, ELCTB.2.3, and ELCTB.3.3, which were eluted with distilled water, 5% MeOH, 10% MeOH, and 20% ACN, respectively, were further injected into preparative HPLC (a preparative Cosmosil 5C18-AR-II column equipped with 250 × 20 mm i.d. and a UV detector (Nacalai Tesque, Inc., Kyoto, Japan) at 221 and 254 nm), to obtain 12 compounds (**1**–**12**). Compounds **1**–**8** were eluted with 5% ACN, compound **11** was eluted with 9% ACN, and compounds **9**, **10,** and **12** were eluted with 23% MeOH. The isolation process of the active compounds from the ELCTB extract are briefly summarized in Figure 1. The chemical structures of the isolated constituents were identified by analysis of their NMR data and a comparison with the reported compounds. The ^1^H and ^13^C-NMR spectra and the 2D-NMR spectra, including NOESY, HMBC, HMQC, and COSY, were measured in MeOH-*d*_4_ on a Bruker AVX NMR spectrometer (Bruker, Karlsruhe, Germany), which operated at 600 MHz for 1–12 h and 150 MHz for ^13^C spectra measurement, and the MeOH-*d*_4_ solvent peak was used as the internal standard (δ_H_ 3.317, δ_C_ 49.1 ppm).

## 4. Conclusions

A total of 12 phenolics (**1**–**12**) were newly isolated and identified from *Euonymus laxiflorus.* Of these, three constituents (**9**), (**11**), and (**12**) were identified as new compounds, while (**1**), (**7**), and (**8**) were identified as new natural products. These 12 compounds and the other 10 compounds (**13**–**22**) obtained from our previous work were evaluated for their antioxidant activity. Notably, 12 compounds (**4**–**6**, **9**, **13**–**15**, **18**–**22**) displayed potent antioxidant activity (FRS50 = 7.8–58.11 µg/mL) compared to α-tocopherol (FRS50 = 23 µg/mL). Among these, two constituents, (**9**) and (**13**), were determined to be new antioxidants. In the anti-NO activity tests, walterolactone A (1a) and B (1b) β-d-glucopyranoside (**13**) was found to have the most effective and comparable activity to that of quercetin, with max inhibition and IC_50_ values of 100%, 1.3 µg/mL and 100%, 1.21 µg/mL, respectively, and this compound was reported to have anti-NO activity for the first time. The crude extract and its major compounds showed no cytotoxicity on normal cells. The results of the current study confirmed that ELCTB was a rich natural source of active phenolics that could be developed as a health food with promising antioxidant, anti-NO, and other beneficial biological activities. The results of this study also contributed to the enrichment of new natural products and the enrichment of novel compounds, as well as the novel biological activity of the constituents isolated from *Euonymus laxiflorus*.

## Figures and Tables

**Table 1:** Antioxidant activity of other MeOH extracts of medicinal plants collected in Dak Lak.

Scientific Name of Medicinal Plants	Part Used	FRS50 (µg/mL)	Ref.
*Euonymus laxiflorus* Champ.	Trunk bark	20.2 ± 0.45	This study
α-Tocopherol *		24.4 ± 0.87	
*Terminalia alata*	Trunk bark	240 ± 20	Nguyen et al., 2016 [5]
*T. bellirica*	Trunk bark	1020 ± 20	Nguyen et al., 2016 [5]
*T. corticosa*	Trunk bark	250 ± 0.00	Nguyen et al., 2016 [5]
l-ascorbic acid *		240 ± 0.00	Nguyen et al., 2016 [5]
*T. nigrovenulosa*	Bark extract	273 ± 3	Nguyen, Q.V. 2017 [10]
*T. nigrovenulosa*	Leaves extract	408 ± 6	Nguyen, Q.V. 2017 [10]

* Commercial antioxidant compounds.

**Table 2:** Evaluation of the antioxidant activity of the isolated compounds.

No.	Isolated compounds	DPPHFRS50 (μg/mL)	Max (%)
**1**	Umbelactone (new natural product)	-	-
**2**	Walterolactone	-	-
**3**	Phenylalanine	-	-
**4**	2-methoxy-4-hydroxyphenol-1-*O*-β-d-glucopyranoside	38.09 ^b^	92 ^a^
**5**	1-β-d-glucopyranosyloxy-3,5-dimethoxy-4-hydroxybenzene	58.11 ^a^	100 ^a^
**6**	3,4-Dihydroxybenzoic acid	16.77 ^e^	95 ^a^
**7**	2-benzoyl myo-inositol (new natural product)	-	-
**8**	1-*O*-Benzoyl-myo-inositol (new natural product)	-	-
**9**	Walterolactone A/B 6-*O*-gallate-β-d-glucopyranoside (new compound)	10.9 ^e,f^	96 ^a^
**10**	Roseoside (6S, 9S)	-	-
**11**	(3R*,6R*)-tetrahydro-6-ethenyl-2,2,6-trimethyl-2H-pyran 3-*O*-α-l-arabinopyranosyl (1→3)-β-d-glucuronopyranosyl (new compound)	UD	25 ^b^
**12**	7-Hydroxy-6,7-dihydro-cis/trans-geraniate, 3-*O*-α-l-arabinopyranosyl (1→6)-β-d-glucupyranosyl (new compound)	UD	20 ^b^
**13**	Walterolactone A (1a) and B (1b) β-d-glucopyranoside	28 ^c,d^	98 ^a^
**14**	Gallic acid	9 ^f^	97 ^a^
**15**	(−)-Gallocatechin	30.73 ^c^	93 ^a^
**16**	1-(4-hydroxyphenyl)-2,3-dihydroxypropan-1-one 3-*O*-β-d-glucopyranoside/or Schweinfurthinol 9-*O*-β-d-glucopyranoside	-	-
**17**	Myo-inositol 1-*O*-3,3-dimethylacrylate/or 1-*O*-(3-methyl)-butenoyl-myo-inositol	-	-
**18**	Leonuriside	27.47 ^c,d^	95 ^a^
**19**	(+)-Catechin	7.10 ^f^	97 ^a^
**20**	Methyl galloate	9.4 ^f^	96 ^a^
**21**	(−)-Catechin	11.5 ^e,f^	96 ^a^
**22**	(3,5-dimethoxy-4-hydroxyphenol)-1-*O*-β-d-(6′-*O*-galloyl)-glucopyranoside	7.8 ^f^	96 ^a^
	α-Tocopherol (commercial antioxidant)	23 ^d^	99 ^a^
	CV (Coefficient of variation, %)	12.33842	7.156336

The purified compounds were tested for their antioxidant activity at concentrations in the range of 0.5–250 μg/mL. The results were presented as the means ± SD of multiple tests (n = 3). The means of max inhibition and FRS50 with different letters in the same column were significantly different based on Duncan’s multiple range test at alpha = 0.01 (UD: unable to determine).
